# Impact of post-COVID conditions on mental health: a cross-sectional study in Japan and Sweden

**DOI:** 10.1186/s12888-022-03874-7

**Published:** 2022-04-04

**Authors:** Kazuki Matsumoto, Sayo Hamatani, Eiji Shimizu, Anton Käll, Gerhard Andersson

**Affiliations:** 1 Research Center for Child Mental Development, Chiba University, Inohana 1-8-1, Chuo-ku, Chiba City, Chiba, Japan; 2 Division of Clinical Psychology, Kagoshima University Hospital, Kagoshima, Japan; 3 Department of Behavioural Sciences and Learning, Linköping University, Linköping, Sweden; 4 Research Center for Child Mental Development, University of Fukui, Fukui, Japan; 5 Department of Cognitive Behavioral Physiology, Graduate School of Medicine, Chiba University, Chiba, Japan; 6 Department of Clinical Neuroscience, Karolinska Institute, Stockholm, Sweden

**Keywords:** Coronavirus disease 2019 (COVID-19), COVID-19 pandemic, Severe acute respiratory syndrome coronavirus 2 (SARS-CoV-2), SARS-CoV-2, Depression, Anxiety, Mental health, Post-COVID conditions

## Abstract

**Background:**

Due to the coronavirus disease 2019 (COVID-19) pandemic, people have undermined their mental health. It has been reported that post-COVID conditions at a certain rate. However, information on the mental health of people with post-COVID conditions is limited. Thus, this study investigated the relationship between post-COVID conditions and mental health.

**Methods:**

Design of the present study was an International and collaborative cross-sectional study in Japan and Sweden from March 18 to June 15, 2021. The analyzed data included 763 adults who participated in online surveys in Japan and Sweden and submitted complete data. In addition to demographic data including terms related to COVID-19, psychiatric symptoms such as depression, anxiety, and post-traumatic stress were measured by using the fear of COVID-19 scale (FCV-19S), Patient Health Questionnaire-9 (PHQ-9), General Anxiety Disorder-7 item (GAD-7), and Impact of Event Scale-Revised (IES-R).

**Results:**

Of the 135 COVID-19 survivors among the 763 total participants, 37.0% (*n* = 50/135) had COVID-19-related sequelae. First, the results of the Bonferroni-corrected Mann Whitney U test showed that the group infected SARS-CoV-2 with post-COVID conditions scored significantly higher than those without one and the non-infected group on all clinical symptom scales (*P* ≤ .05). Next, there was a significant difference that incidence rates of clinical-significant psychiatric symptoms among each group from the results of the Chi-squared test (*P* ≤ .001). Finally, the results of the multivariate logistic model revealed that the risk of having more severe clinical symptoms were 2.44–3.48 times higher among participants with post-COVID conditions.

**Conclusion:**

The results showed that approximately half had some physical symptoms after COVID-19 and that post-COVID conditions may lead to the onset of mental disorders.

**Trial registration:**

The ethics committee of Chiba University approved this cross-sectional study (approval number: 4129). However, as no medical intervention was conducted, a clinical trial registration was not necessary.

## Background

Coronavirus disease 2019 (COVID-2019) caused by severe acute respiratory syndrome coronavirus 2 (SARS-CoV-2) is an ongoing global pandemic. The clinical outcome of COVID-19 ranges from mild respiratory failure to severe disease with high risk of fatality [1]. As of July 26, 2021, the global COVID-19 dashboard shows 194,723,719 coronavirus case patients, 4,167,618 deaths [2]. Recent studies have reported that at least the one in four COVID-19 recoverees suffer long-term impairments such as fatigue and taste/smell disorders [3].

A decrease in the ability of the lungs to diffuse carbon monoxide was often found in patients who recovered from COVID-19 [4], suggesting that respiratory dysfunction may remain after recovery [5]. A recent study by Ballan and colleagues followed up patients three to four months after COVID-19 recovery and reported the following long-term physical impairment: 13 (5.5%) Dyspnea, 12 (5.0%) ageusia, 11 (4.6%) anosmia, 14 (5.9%) arthralgia, 14 (5.9%) myalgia, and 53 (22.3%) limited mobility and 113 (51.6%) decreased vital capacity[6]. Those conditions after COVID-19 have been named as post-COVID conditions. “Experts around the world are working to learn more about short- and long-term health effects associated with COVID-19, who gets them, and why (Centers for Disease Control and Prevention, 2021)” [7].

Mental health problems, as well as physical disabilities, have been observed in people post their recovery from COVID-19. In terms of mental health problems, symptoms of post-traumatic stress disorder (PTSD), depression, or anxiety have been observed in people who recovered from COVID-19 [6, 8]. Anxiety and depression may be common six months after COVID-19 recovery, and people who present a more serious condition in the acute phase of COVID-19 are more likely to develop symptoms of depression and anxiety [9]. Those long-term effects are in line with the previous severe acute respiratory syndrome (SARS) [10]. It has been reported that the physical impairment and mental disorders occur after COVID-19, though the relationship between them has been not insufficiently investigated. Physical disability can lead to depression [11–15], and people with disabilities are about three times riskier to have depression than people without disabilities [16–19]. As mentioned above, many people have impaired physical functioning after recovering from COVID-19. Respiratory disorders are particularly likely to remain, and they may cause dysfunction in daily life [20]. However, to the best of our knowledge, an investigation into whether post-COVID conditions causes mental disorders has not been undertaken. Identifying COVID-19 patients who are most likely to need assistance due to physical and psychiatric symptoms can have implications for long-term support policies for COVID-19-infected individuals.

Evidence from previous studies suggest that COVID-19 infection control measures such as lockdown may be related to the mental health of citizens. Citizens are at an increased risk of mental disorders, such as depression, in countries that have implemented lockdowns [21–23]. A systematic review of depression outcomes in 33 countries found that the prevalence of clinically significant depressive symptoms was significantly lower in countries where the government quickly implemented strict policies [24]. However, this review did not include data from Japan; Swedish findings were included. Japan and Sweden have made their own policy decisions without lockdown [25, 26]. During the COVID-19 pandemic, knowledge with regard to the mental health of general citizens and the citizens of these countries entrusted with infection control measures is limited. Therefore, studying the characteristics of the mental health of citizens in Japan and Sweden during the COVID-19 pandemic may facilitate an understanding of the impact of pandemic policy making on people's mental health.

The objectives of the present study were to investigate the prevalence of post-COVID conditions, and clinical associations between post-recovery physical function and psychosocial disorders in individuals who had been infected with SARS-CoV-2. We report the results of a research for post-COVID conditions and their effects on the mental health of study participants in Japan and Sweden.

## Methods

### Study design

We conducted a cross-sectional study in Japan and Sweden from March 18 to June 15, 2021 and collected the data through an online survey. The only eligibility criterion was that the age of the participants should be at least 18 years old. Data collection was outsourced to Asmark companies in Japan and Prolific in Sweden, and data collection was carried out through each company's online research platform. Each company asked pooled participants to participate in the study ― that is, to respond online. Participants answered the questions by accessing the websites. They were informed in the first half of the questionnaire that the survey content included information about the COVID-19 hospitalization experience and post-COVID conditions, so participants could withdraw their participation, if they wished, depending on the degree of psychological distress. A small monetary compensation was paid as a reward to the participants through the research company.

The study was planned and designed by researchers in Japan and Sweden according to the STROBE statement [27]. The protocol for the current observational study was reviewed and approved by the Chiba University Graduate School of Medicine Ethics Review Committee (approval number 4129). The online survey was written in the native languages of Japan and Sweden, and it took about 20 min to complete.

### Setting

We recruited 763 participants from Japan and Sweden. Of the participants, 135 had been infected with COVID-19 and 628 had never been infected with COVID-19. Data were collected in Japan from March 18 to 22, 2021 and in Sweden from April 5 to June 15, 2021 (e.g., a slight difference in time).

### Measures

#### Demographic data

We collected the following background information about the of the participants: age, gender (woman, male and prefer not to say), race, occupation (regular, non-regular, unemployed, college student), family structure (living with family, living alone, sharing a house with someone other than the family), academic background, financial situation (household annual income of less than 4270,000 JPY (about 38,366 dollars) or more/ 380,000 SEK (about 44,408 dollars or more), having enough savings to live for about half a year if you lose your current job (yes, no), living area (up to prefecture), history of mental illness (depression, bipolar disorder, schizophrenia, anxiety, PTSD, obsessive–compulsive disorder, panic disorder, eating disorder, substance use disorder, etc.; free description), presence or absence of history of physical illness (high blood pressure, asthma, diabetes, etc.; free description).

#### Data on COVID-19

We collected information about the participants’ experience with COVID-19 (presence of infection, time of infection, time required for recovery, acute symptoms, physical sequelae, and vaccination). Participants were asked about symptoms related to COVID-19 and responded “yes” or “no” to the following items: heat, cough, fatigue/tiredness, dyspnea, olfaction disorder, dysgeusia, increased sputum, chest pain, joint pain, muscle pain, headache, hair loss, insomnia, anxiety, depression, and sore throat. If they experienced any symptoms related to COVID-19 other than the items mentioned above, the participants were free to mention them.

#### Criteria for mental disorders

Mental health was evaluated by four psychological measure scales to assess fear of COVID-19, depression, general anxiety, post-traumatic stress. The Fear of COVID-19 Scale (FCV-19S) is a self-rating scale with seven items that can quantify the fear of COVID-19. The total scores range from 7 to 35 points, and the higher the score, the stronger the fear of COVID-19 [28, 29]. Patient Health Questionnaire-9 (PHQ-9), with 9 items, is a self-rating scale to assess severity of depression. The total score of PHQ-9 shows 1 to 4 points are mild, 5 to 9 points are mild, 10 to 14 points are moderate, 15 to 19 points are moderate to severe, and 20 to 27 points are severe [30, 31]. General Anxiety Disorder-7 -item (GAD-7), with 7 items, is a self-rating scale to assess severity of general anxiety. The total score of GAD-7 shows o to 4 points are minimal anxiety, 5 to 9 points are mild, 10 to 14 points are moderate, 15 to 21 points are severe [32, 33]. Impact of Event Scale-Revised (IES-R) is a self-rating scale for measuring traumatic stress symptoms. This scale consists of 8 items of invasion symptoms, 8 items of avoidance symptoms, and 6 items of hypervigilance symptoms, for a total of 22 items [34, 35]. We used cutoffs as the criterion for the incidence of each clinical measure. The cutoff for FCV-19S was 18 points or more [29], 10 points or more for PHQ-9 [31], 10 points or more for GAD-7 [33], and 25 points or more for the IES-R [35].

### Dealing with bias

People who have been recovering for some time may not be able to remember the early aftereffects of recovery following a COVID-19 infection. To address this potential recall bias, we asked about sequelae and mental health at the time of the survey. Because the data were collected via the internet, there may be a selection bias as the survey only reach those who have access to the internet and are interested in health. To address this selection bias, we conducted the survey in two countries (Japan and Sweden) in which internet usage is extremely high. Internet usage rates have been over 90% in both countries for a long time [36]. Although there are some regulations in both countries, strict lockdown has never been implemented since the onset of the COVID-19 pandemic. Therefore, we assumed that ordinary people in both countries 2021 would be interested in a survey on COVID-19.

### Sample size

The assumed effect size calculated by G*Power was 0.30, two-tail. The power was set at 0.80 and the significance level at 0.05. The sample size needed to obtain sufficient power by the* F* test was estimated to be 37 people in each group (non-infected group, infected without post-COVID conditions, and infected with one). To collect at least 74 infected people, the final total sample size was set to 800 after taking into account the proportion of infected people and missing data.

### Statistical analysis

We used SPSS Version 26 (IBM Corporation, Armonk, NY, USA) for four statistical analyses. A two-sided *P* value of < 0.05 was considered statistically significant, and *p*-values for the Mann–Whitney U test were deemed statistically significant at the Bonferroni corrected *p* < 0.05. Firstly, we performed the Kruskal–Wallis tests among the uninfected people, the infected people with post-COVID conditions, and those without sequelae. Secondly, we conducted the Bonferroni-corrected Mann–Whitney U test as Post hoc comparisons to verify which group had a significant difference. Thirdly, we conducted a Chi-squared test to compare the three groups mentioned above and verify if there was a significant difference in the proportion of people at high risk of clinically significant mental illness. Finally, we conducted logistic regression analyses to evaluate the impact of post-COVID conditions on mental health. In a logistic regression model, we assessed whether the nine variables (age, country, gender, mental illness, physical illness, days to recovery, hospitalization, post-COVID conditions, and ventilator) were associated with determinants of clinical symptom incidence. In the logistic regression model, we also calculated the adjusted odds ratio (OR) with a 95% confidence interval (CI) for the risk of clinically significant symptoms of depression, anxiety, and post-traumatic stress for the participants with post-sequelae after COVID conditions-19.

## Results

### The demographic data of participants

Table 1 presents demographic data. There were 269 women (35.3%), 487 men (63.8%), and 7 who preferred not to state their gender (0.9%). Of the 763 participants, 135 had developed COVID-19 and 628 had not. Table 2 shows the history of mental illness and physical illness, presence or absence of hospitalization, presence or absence of a respirator, number of days until recovery, symptoms of COVID-19, and post-COVID conditions. There were 36 (46.0%) Japanese and 14 (31.3%) Swedish participants who still had post-COVID conditions. Among the Japanese, the main post-COVID conditions were dysgeusia (*n* = 11, 30.6%), fatigue, tiredness (*n* = 10, 27.8%), olfactory dysfunction (*n* = 7, 19.4%), chest pain (*n* = 6, 16.7%), coughing (*n* = 6, 16.7%), and palpitations (*n* = 5, 13.9%). Among the Swedes, the main post-COVID conditions were fatigue, tiredness (*n* = 9, 64.3%), olfactory dysfunction (*n* = 5, 35.7%), fever (*n* = 3, 21.4%).

**Table 1:** Demographic data of the participants

	**Overview**			**Japanese**			**Swedish**		
	Total (*n* = 763)	Non-infected people (*n* = 628)	Infected people (*n* = 135)	Total (*n* = 387)	Non-infected people (*n* = 300)	Infected people (*n* = 87)	Total (*n* = 376)	Non-infected people (*n* = 328)	Infected people (*n* = 48)
	n (%)	n (%)	n (%)	n (%)	n (%)	n (%)	n (%)	n (%)	n (%)
**Gender**									
Women	269 (35.3)	402 (64.0)	50 (37.0)	138 (35.7)	107 (35.7)	31 (35.6)	131 (34.8)	112 (34.1)	19 (39.6)
Men	487 (63.8)	219 (34.9)	85 (63.0)	249 (64.3)	193 (64.3)	56 (64.4)	238 (63.3)	209 (63.7)	29 (60.4)
Prefer not to say	7 (0.9)	7 (1.1)	-	-	-	-	7 (1.9)	7 (2.1)	-
**Age** ^a^	36.7 ± 15.1	36.4 ± 15.0	38.3 ± 15.7	43.9 ± 16.6	43.9 ± 16.8	43.8 ± 15.9	29.3 ± 8.6	29.5 ± 8.6	28.2 ± 9.1
**Education**									
Junior high school graduate	36 (4.7)	30 (4.8)	6 (4.4)	10 (2.6)	8 (2.7)	2 (2.3)	26 (6.9)	22 (6.7)	4 (8.3)
High school graduate	274 (35.9)	231 (36.8)	43 (31.9)	98 (25.3)	81 (27.0)	17 (19.5)	176 (46.8)	150 (45.7)	26 (54.2)
Vocational school/junior college graduate	83 (10.9)	68 (10.8)	15 (11.1)	53 (13.7)	40 (13.3)	13 (14.9)	30 (8.0)	28 (8.5)	2 (4.2)
University graduate or above	370 (48.5)	299 (47.6)	71 (52.6)	226 (58.4)	171 (57.0)	55 (63.2)	144 (38.3)	128 (39.0)	16 (33.3)
**Household income**									
≤ 4270,000 JPY or ≤ 380,000 SEK	367 (48.1)	308 (49.0)	59 (43.7)	162 (41.9)	130 (43.3)	32 (36.8)	205 (54.5)	178 (54.3)	27 (56.3)
>4270,000 JPY or > 380 000 SEK	396 (51.9)	320 (51.0)	76 (56.3)	225 (58.1)	170 (56.7)	55 (63.2)	171 (45.5)	150 (45.7)	21 (43.8)
**Worker status**									
Full time	371 (48.6)	291 (46.3)	80 (59.3)	208 (53.7)	148 (49.3)	60 (69.0)	163 (43.4)	143 (43.6)	20 (41.7)
Part time	76 (10.0)	63 (10.0)	13 (9.6)	59 (15.2)	48 (16.0)	11 (12.6)	17 (4.5)	15 (4.6)	2 (4.2)
Unemployed	155 (20.3)	137 (21.8)	18 (13.3)	88 (22.7)	75 (25.0)	13 (14.9)	67 (17.8)	62 (18.9)	5 (10.4)
Student	161 (21.1)	137 (21.8)	24 (17.8)	32 (8.3)	29 (9.7)	3 (3.4)	129 (34.3)	108 (32.9)	21 (43.8)
**Living status**									
Alone	202 (26.5)	165 (26.3)	37 (27.4)	94 (24.3)	72 (24.0)	22 (25.3)	108 (28.7)	93 (28.4)	15 (31.3)
With family or partner	546 (71.6)	451 (71.8)	95 (70.4)	290 (74.9)	226 (75.3)	64 (73.6)	256 (68.1)	225 (68.6)	31 (64.6)
With others	15 (2.0)	12 (1.9)	3 (2.2)	3 (0.8)	2 (0.7)	1 (1.1)	12 (3.2)	10 (3.0)	2 (4.2)
**Diagnosed mental disorders**									
Yes	226 (29.6)	168 (26.8)	58 (43.0)	92 (23.8)	53 (17.7)	39 (44.8)	134 (35.6)	115 (35.1)	19 (39.6)
No	537 (70.4)	460 (73.2)	77 (57.0)	295 (76.2)	247 (82.3)	48 (55.2)	242 (64.4)	213 (64.9)	29(60.4%)
**Physical illness**									
Yes	192 (25.2)	138 (22.0)	54 (40.0)	115 (29.7)	68 (22.7)	47 (54.0)	77 (20.5)	70 (21.3)	7 (14.6)
No	571 (74.8)	490 (78.0)	81 (60.0)	272 (70.3)	232 (77.3)	40 (46.0)	299 (79.5)	258 (78.7)	41 (85.4)
**Taken vaccine for COVID-19**									
Yes	78 (10.2)	37 (5.9)	41 (30.4)	41 (10.6)	4 (1.3)	37 (42.5)	37 (9.8)	33 (10.1)	4 (8.3)
No	685 (89.8)	591 (94.1)	94 (69.6)	346 (89.4)	296 (98.7)	50 (57.5)	339 (90.2)	295 (89.9)	44 (91.7)
**Infected with SARS-CoV-2**									
Yes	135 (17.7)	-	135 (17.7)	87 (22.5)	-	87 (22.5)	48 (12.8)	-	48 (12.8)
No	628 (82.3)	628 (82.3)	-	300 (77.5)	300 (77.5)	-	328 (87.2)	328 (87.2)	-

^a^Mean ± SD

**Table 2:** Demographic data of participants infected with SARS-CoV-2

	**Overview**			**Japanese**			**Swedish**		
	Non-infected people (*n* = 628)	Without post-COVID conditions (*n* = 85)	With post-COVID conditions (*n* = 50)	Non-infected people (*n* = 300)	Without post-COVID conditions (*n* = 51)	With post-COVID conditions (*n* = 36)	Non-infected people (*n* = 328)	Without post-COVID conditions (*n* = 34)	With post-COVID conditions (*n* = 14)
	n (%)	n (%)	n (%)	n (%)	n (%)	n (%)	n (%)	n (%)	n (%)
**Age** ^a^	36.4 ± 15.0	37.3 ± 16.3	39.9 ± 14.7	43.9 ± 16.8	43.9 ± 16.8	43.8 ± 14.6	29.5 ± 8.6	27.4 ± 9.0	30.0 ± 9.4
**Diagnosed mental illness (yes)**	168 (26.8)	29 (34.1)	29 (58.0)	53 (17.7)	18 (35.3)	21 (58.3)	115 (35.1)	11 (32.4)	8 (57.1)
Depression	129 (20.5)	15 (17.6)	20 (40.0)	40 (13.3)	7 (13.7)	14 (38.9)	89 (27.1)	8 (23.5)	6 (42.9)
Bipolar disorder or schizophrenia	12 (1.9)	4 (4.7)	8 (16.0)	8 (2.7)	2 (3.9)	8 (22.2)	4 (1.2)	2 (5.9)	-
Anxiety disorder	65 (10.4)	9 (10.6)	13 (26.0)	14 (4.7)	6 (11.8)	11 (30.6)	51 (15.5)	3 (8.8)	2 (14.3)
PTSD	11 (1.8)	3 (3.5)	4 (8.0)	2 (0.7)	3 (5.9)	4 (11.1)	9 (2.7)	-	-
OCD	10 (1.6)	2 (2.4)	6 (12.0)	1 (0.3)	1 (2.0)	6 (16.7)	9 (2.7)	1 (2.9)	-
Panic disorder	27 (4.3)	6 (7.1)	10 (20.0)	9 (3.0)	5 (9.8)	9 (25.0)	18 (5.5)	1 (2.9)	1 (7.1)
Eating disorders	16 (2.5)	3 (3.5)	6 (12.0)	1 (0.3)	3 (5.9)	4 (11.1)	15 (4.6)	-	2 (14.3)
Substance Use Disorder	4 (0.6)	3 (3.5)	6 (12.0)	-	3 (3.5)	5 (13.9)	4 (1.2)	-	1 (7.1)
ADHD	5 (0.8)	1(1.2)	1 (2.0)	-	-	-	5 (1.5)	1 (2.9)	1 (7.1)
ADD	1 (0.2)	-	-	-	-		1 (0.3)		
ASD	5 (0.8)	-	-	1 (0.3)	-	-	4 (1.2)	-	-
Schizophrenia personality disorder	1 (0.2)	-	-		-	-	1 (0.3)		
Borderline personality disorder	1 (0.2)	-	-		-	-	1 (0.3)		
**Physical illness (yes)**	138 (22.0)	24 (28.2)	30 (60.0)	68 (22.7)	20 (39.2)	27 (75.0)	70 (21.3)	4 (11.8)	3 (21.4)
High blood pressure	51 (8.1)	15 (17.6)	22 (44.0)	43 (14.3)	14 (27.5)	22 (61.1)	8 (2.4)	1 (2.9)	-
Asthma	48 (7.6)	6 (7.1)	12 (24.0)	13 (4.3)	3 (5.9)	9 (25.0)	35 (10.7)	3 (8.8)	3 (21.4)
Diabetes	23 (3.7)	7 (8.2)	10 (20.0)	15 (5.0)	6 (11.8)	10 (27.8)	8 (2.4)	1 (2.9)	-
Cancer		2 (2.4)		-	2 (4.0)	-	-	-	-
Renal failure		1 (1.2)	-	-	1 (2.0)	-	-	-	-
Chronic nephritis		1 (1.2)	-	-	1 (2.0)	-	-	-	-
Other	28 (4.5)			8 (2.6)			20 (6.1)		
**Hospitalization (yes)**	-	23 (27.1)	25 (50.0)	-	23 (45.1)	25 (69.4)	-	-	-
Ventilator	-	10 (11.8)	13 (26.0)	-	10 (19.6)	13 (36.1)	-	-	-
Days to recovery ^b^	-	23.8 ± 30.4	35.2 ± 39.1	-	29.27 ± 36.1	35. 2 ± 33.6	-	15.6 ± 16.1	35.1 ± 52.2
**Infectious symptoms (yes)**	-	79 (82.4)	50 (100.0)	-	39 (76.5)	36 (100)	-	34 (100.0)	14 (100.0)
Heat	-	45 (52.9)	38 (76.0)	-	27 (52.9)	27 (75.0)	-	18 (52.9)	11 (78.6)
Cough	-	29 (34.1)	24 (48.0)	-	12 (23.5)	17 (47.2)	-	17 (50.0)	7 (50.0)
Fatigue, tiredness	-	39 (45.9)	26 (52.0)	-	14 (27.5)	16 (44.4)	-	25 (73.5)	10 (71.4)
Dyspnea	-	5 (5.9)	11 (22.0)	-	3 (5.9)	10 (27.8)	-	2 (5.9)	1 (7.1)
Olfactory dysfunction	-	13 (15.3)	19 (38.0)	-	3 (5.9)	11 (30.6)	-	10 (29.4)	8 (57.1)
Dysgeusia	-	20 (23.5)	17 (34.0)	-	7 (13.7)	11 (30.6)	-	13 (38.2)	6 (42.9)
Increased sputum	-	8 (9.4)	9 (18.0)	-	2 (3.9)	5 (13.9)	-	6 (17.6)	4 (28.6)
Chest pain	-	5 (5.9)	10 (20.0)	-	3 (5.9)	8 (22.2)	-	2 (5.9)	2 (14.3)
Joint pain	-	11 (12.9)	16 (32.0)	-	3 (5.9)	7 (19.4)	-	8 (23.5)	9 (64.3)
Muscle pain	-	10 (11.8)	13 (26.0)	-	1 (2.0)	8 (22.2)	-	9 (26.5)	5 (35.7)
Headache	-	23 (27.1)	16 (32.0)	-	3 (5.9)	9 (25.0)	-	20 (58.8)	7 (50.0)
Palpitations	-	1 (1.2)	9 (18.0)	-	1 (2.0)	5 (13.9)	-	-	4 (28.6)
Hair loss	-	1 (1.2)	3 (6.0)	-	1 (2.0)	3 (8.3)	-	-	-
Sore throat	-	4 (4.7)	3 (6.0)	-	2 (4.0)	2 (5.6)	-	2 (5.9)	1 (7.1)
Urination disorder	-	1 (1.2)	-	-	1 (2.0)	-	-	-	-
Nasal congestion	-	-	1 (2.0)	-	-	1 (2.8)	-	-	-
Chills	-	1 (1.2)	-	-	1 (2.0)	-	-	-	-
Diarrhea	-	-	1 (2.0)	-	-	-	-	-	1 (7.1)
Nosebleed	-	1 (1.2)	-	-	-	-	-	1 (2.9)	-
Herpes labialis	-	1 (1.2)	-	-	-	-	-	1 (2.9)	-
Common cold	-	1 (1.2)	-	-	-	-	-	1 (2.9)	-
Respiratory tract infection	-	1 (1.2)	-	-	-	-		1 (2.9)	-
Insomnia	-	4 (4.7)	4 (8.0)	-	1 (2.0)	2 (5.6)	-	3 (8.8)	2 (14.3)
Anxiety	-	5 (5.9)	6 (12.0)	-	4 (7.8)	4 (11.1)	-	1 (2.9)	2 (14.3)
Depression	-	4 (4.7)	5 (10.0)	-	3 (5.9)	3 (8.3)	-	1 (2.9)	2 (14.3)
**Post-COVID conditions (yes)**	-	-	-	-	-		-	-	
Heat	-	-	7 (14.0)	-	-	4 (11.1)	-	-	3 (21.4)
Cough	-	-	8 (16.0)	-	-	6 (16.7)	-	-	2 (14.3)
Fatigue, tiredness	-	-	19 (38.0)	-	-	10 (27.8)	-	-	9 (64.3)
Dyspnea	-	-	5 (10.0)	-	-	4 (11.1)	-	-	1 (7.1)
Olfactory dysfunction	-	-	12 (24.0)	-	-	7 (19.4)	-	-	5 (35.7)
Dysgeusia	-	-	13 (26.0)	-	-	11 (30.6)	-	-	2 (14.3)
Increased sputum	-	-	6 (12.0)	-	-	4 (11.1)	-	-	2 (14.3)
Chest pain	-	-	7 (14.0)	-	-	6 (16.7)	-	-	1 (7.1)
Joint pain	-	-	5 (10.0)	-	-	3 (8.3)	-	-	2 (14.3)
Muscle pain	-	-	6 (12.0)	-	-	4 (11.1)	-	-	2 (14.3)
Headache	-	-	5 (10.0)	-	-	3 (8.3)	-	-	2 (14.3)
Palpitations	-	-	6 (12.0)	-	-	5 (13.9)	-	-	1 (7.1)
Hair loss	-	-	5 (10.0)	-	-	4 (11.1)	-	-	1 (7.1)
Sore throat	-	-	1 (2.0)	-	-	1 (2.8)	-	-	-
Feeling like stinging in your left hand	-	-	1 (2.0)	-	-	-	-	-	1 (7.1)
**Clinical scales**							-	-	
FCV-19S ≥ 18	269 (42.8)	39 (45.9)	36 (72.0)	191 (63.7)	35 (68.6)	32 (88.9)	78 (23.8)	4 (11.8)	4 (28.6)
PHQ ≥ 10	190 (30.3)	25 (29.4)	28 (56.0)	72 (24.0)	17 (33.3)	22 (61.1)	118 (36.0)	8 (23.5)	6 (42.9)
GAD ≥ 10	105 (16.7)	13 (15.3)	20 (40.0)	45 (15.0)	11 (21.6)	16 (44.4)	60 (18.3)	2 (5.9)	4 (28.6)
IES-R ≥ 25	192 (30.6)	35 (41.2)	33 (66.0)	95 (31.7)	25 (49.0)	26 (72.2)	97 (29.6)	10 (29.4)	7 (50.0)

^a^Mean ± SD

*ADD* attention deficit disorder; *ADHD* attention deficit hyperactivity disorder, *ASD* autism spectrum disorder, *COVID-19* coronavirus disease 2019, *IES-R* impact of events scale-revised, *FCV-19S* fear of COVID-19 scales, *GAD-7* generalized anxiety disorder-7 items, *OCD* obsessive–compulsive disorder, *PHQ-9* patients health questionnaire-9 items, *PTSD* post-traumatic stress disorder

### Results of mental health between the groups

**Fig. 1:**
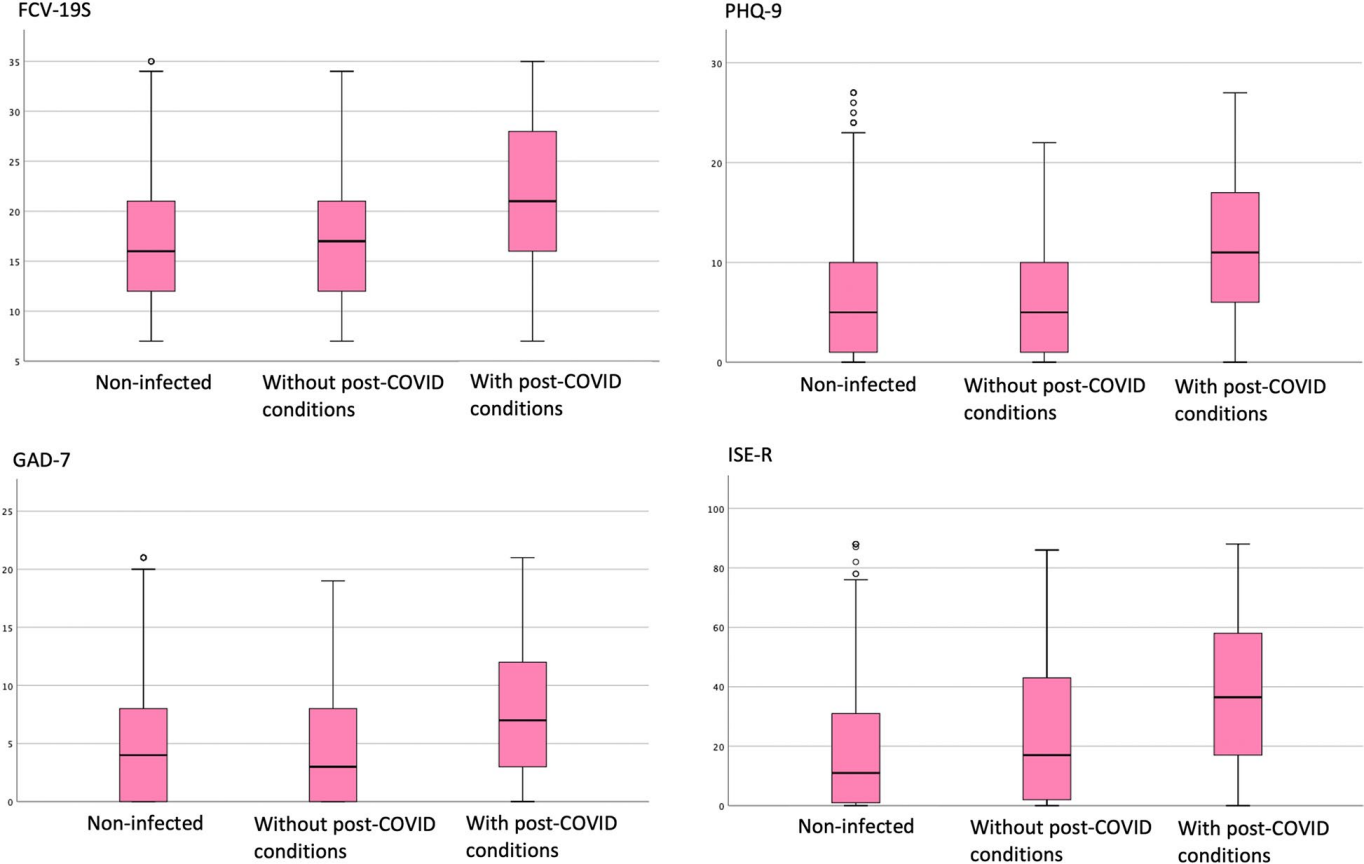
The results of the Kruskal–Wallis tes**t**

Figure 1 show that the results of the Kruskal–Wallis-test, there were significant differences on the following scales of clinical symptoms on mental disorders: FCV-19S (H (2) = 20.5, *P* ≤ 0.001), PHQ-9 (H (2) = 17.0, *P* ≤ 0.001), GAD-7 (H (2) = 21.2, *P* ≤ 0.001), and IES-R (H (2) = 26.4, *P* ≤ 0.001). The results of the Bonferroni-corrected Mann Whitney U test indicated that the group that had developed COVID-19 with post-COVID conditions showed significantly higher scores on all scales (*P* ≤ 0.05) than the group without one and the group that had not developed COVID-19. There was no significant difference between infected group without post-COVID conditions and non-infected groups.

**Fig. 2:**
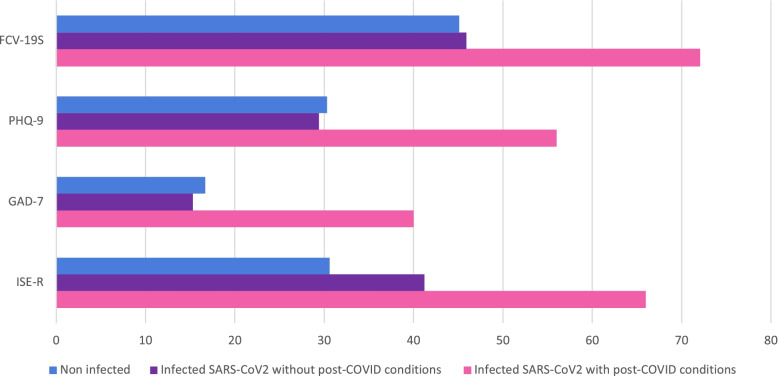
The incidence of clinically significant psychiatric symptoms

Figure 2 shows the incidence of clinically significant psychiatric symptoms in each group. For clinically significant syndrome of COVID-19-related anxiety, depression, general anxiety, and PTSD, the proportion of the participants, who exceeded the cut-off on each clinical symptom rating scale, were significantly high in the group that had developed COVID-19 with post-COVID conditions. Regarding the incidence of clinically significant psychiatric symptoms between the three groups, the results of the Chi-squared test showed a significant difference in all of the above scales (*P* ≤ 0.001).

### Impact of post-COVID conditions on mental health

The risk of developing mental disorders with participants that had developed COVID-19 with post-COVID conditions was at least twice as high as in the participants without one: FCV-19S: 2.93 (95% CI:1.09–7.91); PHQ-9: 2.96 (95% CI: 1.29–6.79); GAD-7: 3.48 (95% CI: 1.30–9.31); ISE-R: 2.44 (95% CI: 1.10–5.43) (see Table 3).

**Table 3:** The results of the logistic regression model

Variable		*β*	*SE*	Odd ratios	95% CI		*P-value*
Dependent	Independent						
FCV-19S	Country	3.18	0.54	24.15	8.34	69.95	< .001
	post-COVID conditions	1.08	0.51	2.93	1.09	7.91	.034
	Gender	1.18	0.52	3.27	1.19	8.98	.022
	Mental illness	1.01	0.49	2.75	1.09	7.91	.041
PHQ-9	Country	1.29	0.50	3.64	1.37	9.70	0.01
	post-COVID conditions	1.09	0.42	2.96	1.29	6.79	.010
	Gender	1.20	0.45	3.32	1.39	7.97	.007
	Mental illness	0.86	0.41	2.36	1.06	5.24	.036
	Age	-0.04	0.02	0.96	0.93	0.99	.009
GAD-7	Country	2.07	0.63	7.89	2.29	27.14	.001
	post-COVID conditions	1.25	0.50	3.48	1.30	9.31	.013
	Gender	0.94	0.54	2.57	0.90	7.35	.079
	Mental illness	1.56	0.51	4.75	1.76	12.81	.002
	Age	-0.06	0.02	0.94	0.90	0.98	.003
IES-R	Country	1.48	0.47	4.39	1.75	11.0	.002
	post-COVID conditions	0.89	0.41	2.44	1.10	5.43	.028
	Mental illness	0.78	0.40	2.18	1.01	4.73	.048
	Age	-0.04	0.02	0.97	0.94	0.99	.015

*CI* confidence interval,*FCV-19S*fear of COVID-19 scale,*GAD-7* deneralised anxiety disorder 7-item scale,*IES-R* impact of event scale-revised,*PHQ-9* patient health questionnaire 9- item scale, *SE* standard error

## Discussion

### The principal findings

The objective of the present study was to identify post-COVID conditions and investigate the relationship between post-COVID conditions and mental health status. We conducted an online survey in two countries and collected valid responses from a total of 763 participants, including 135 with a history of COVID-19. Of the 135 COVID-19 infected participants, 37.0% (*n* = 50/135) had some post-COVID conditions. The major post-COVID conditions reported were fatigue/tiredness (*n* = 19/50, 38.0%), dysgeusia (*n* = 13/50, 26.0%), olfactory dysfunction (*n* = 12/50, 24.0%). The COVID-19-infected respondents showed greater incidence of all mental health symptoms investigated in this study, including symptoms of COVID-19-related anxiety, depression, generalized anxiety, and post-traumatic stress. Greater impairment of mental health was observed in the participants who had developed COVID-19 with post-COVID conditions than those without one. Furthermore, our results suggest that mental health was impaired in the presence of post-COVID conditions.

### The implications of the findings

The group that developed COVID-19 has worse mental health than the group not infected with SARS-CoV-2. In the group with COVID-19 experience, 43.0% (*n* = 58/135) people reported having some sort of mental disorder. In addition, in the COVID-19 experience group, the percentage of people who exceeded the cutoff was 39.3% (*n* = 53/135) for depression, 24.4% (*n* = 33/135) for generalized anxiety, and 50.4% (*n*= 68/135) for PTSD. The participants infected SARS-CoV-2 had significant psychiatric symptoms compared to the participants had not be infected. The results are consistent with those of (i) a cohort study of inpatients with COVID-19 for a one-month period in Helsinki [37], (ii) a British community cohort study [38], and (iii) an American electronic health record network cohort study [39]. The latest systematic review shows that the incidence of depressive symptoms is 10.0–68.5%, that of clinically significant anxiety is 5.0–55.2%, acute and post-traumatic stress is 7.0–36.4%, and fatigue is 12.7–88.6% [40]. The evidence indicates that SARS-CoV-2 infection may increase the risk of developing mental disorders such as depression, anxiety disorders, and PTSD [39].

The incidence of long-term COVID-19 health hazards was 37.0% (*n* = 50/135 after COVID-19 among Japanese and Swedish participants in the present study; relatively common post-COVID conditions were fatigue/tiredness (*n* = 19/50, 38.0%), dysgeusia (*n* = 13/50, 26.0%), olfactory dysfunction (*n* = 12/50, 24.0%). The other frequently observed post-COVID conditions in our study is cough (*n*= 8/50, 16.0%). These results are consistent with previous studies in which most patients had abnormal respiratory function at 3 months [41], meaning that patients with acute symptoms of COVID-19 that were severe enough to require occasional ventilation may have impaired long-term respiratory function. Fatigue/tiredness was reported at 38.0% (*n*= 19/50) in infected respondents in this study. Fatigue after COVID-19 may be associated with lung, cardiovascular, liver, kidney, cognitive, and neural function in some SARS-CoV-2 infected individuals experiencing serious complications during the acute phase [42–46].

Furthermore, the logistic regression analysis showed that the participants with post-COVID conditions were at a higher risk of developing mental disorders than those without one. Historically, non-major symptoms of infectious diseases have been neglected; a COVID-19 long-hauler reported that his medical doctor/practitioners disbelieved his physiological distress [47]. The results from our study can provide practitioners and clinicians with evidence of late-onset long-term symptoms in SARS-CoV-2 infected individuals and suggestions for the risk of subsequent development of mental disorders. The findings also contain information that will help medical policymakers make decisions, highlighting the need to provide long-term medical-psychosocial support services to patients infected by SARS-CoV-2.

Of the participants who had not developed COVID-19, 30.3% exceeded the PHQ-9 cutoff. This result suggests increasing risk of developing clinically depression in the citizens as a whole during pandemic. Our finding is similar to the results of the latest meta-analysis that reported that the prevalence of depression during the COVID-19 pandemic was 25% (ranging from 7.45% to 48.3%) [48]. Therefore, although caution is required in the interpretation due to the non-random sampling procedure, our results indicate that clinically significant depressive symptoms in the general population in Japan and Sweden might be common during the COVID-19 pandemic. In the United States, Ettman et al. (2020) reported that the prevalence of clinical significantly depressive symptoms (the total PHQ-9 score ≥ 10) in the general population increased from 8.5% to 27.8% during the COVID-19 pandemic [49]. Without public health crises such as the COVID-19 pandemic, the ratio of PHQ-9 scores above this threshold for the general adult population has been approximately 6%: 6.5% in South Korea, 5.7% in Japan, and 6.4% in 27 European countries [50]. A recent meta-analysis also suggested a one-year prevalence of depression of 7.2% in 30 countries around the world [51]. The results of the study and the global prevalence of depression demonstrate that people's mental health is compromised during a pandemic, even without lockdown, as seen in Japan and Sweden. Limitations of interpersonal interaction, leisure, and other activities owing to the COVID-19 pandemic, may be associated with exacerbation of depressive symptoms [52, 53].

The results of this study suggest that many COVID-19 survivors have a long-term psychical impairment. A cohort study in Sweden reported that one out of five inpatients required rehabilitation intervention even five months after discharge [54]. These findings indicate that the next direction should be building a system that provides rehabilitation interventions to an unprecedented number of people suffering physical impairments, such as post-COVID conditions. Furthermore, our results show that people's mental health deteriorates with or without a COVID-19 experience during the pandemic. Therefore, the decision-maker should introduce or recommend an intervention format for people with support needs due to mental health problems. Cognitive-behavioral therapy (CBT) may be a promising approach even during the COVID-19 pandemic. Cognitive-behavioral therapy is a highly effective psychotherapy for major depressive disorder, anxiety disorders, and PTSD [55, 56]. Internet-Based CBT (ICBT) via videoconference or web-based program also is as effective as face-to-face CBT [57–59]. Remote treatment such as ICBT does not require people to visit the hospital and may help reduce the risk of SARS-CoV-2 infection. ICBT is also a cost-effective treatment approach that optimizes relatively few therapist resources [60]. ICBT is employed widely in Sweden [61], but not in Japan [62]. In addition, some recent clinical trials have suggested that ICBT is feasible and effective for anxiety disorders in Japan [63, 64]. Therefore, Japan should accelerate efforts to introduce and implement this intervention as broadly as Sweden.

### Strengths, limitations, and directions for future research

The present research has four strengths. First, our results suggest that post-COVID conditions represent a risk for mental illness, and they have deepened our knowledge of the relationship between post-COVID conditions and mental disorders. Second, participants in the present study also included data on patients with relatively mild COVID-19 who have not been hospitalized. Thus, the findings of this study may be applicable to patients with differing severities of COVID-19. Third, the present study population was diverse because this study was conducted in two geographical regions, Eastern Asia and Northern Europe (Table 1). The fourth strength is that the participants have not been intentionally exposed and treated by a particular medical facility because the present research was an online study of cross-sectional study design (Wang and Cheng, 2020) [65].

The limitations of the present study include the nature of the sample, the test format, and accessibility. First, in the survey most respondents were adults in their 20 s and 30 s in the Swedish data set. In contrast, there were few respondents aged 50 years or older. Elderly people, who often have chronic physical illnesses, are a group that demonstrate more serious symptoms of COVID-19 [66, 67], which can be more detrimental to post-COVID conditions and mental health [68]. In future studies, conducting research with a larger sample size and analyzing them by age group is necessary. Second, all mental health measures were rated on a self-rating scale. Although the data collected was well-validated and the severity of clinical symptoms was credible, clinical symptoms alone do not confirm any mental disorders. In the future, cross-sectional populations should be assessed using telephone interview and using diagnostic classification tests conducted in semi-structured interviews by trained clinicians. The third limitation was that the population may have belonged to a relatively wealthy social class with a high degree of education, information and communication technology (ICT) literacy, and possession of an internet environment and devices. Populations who have access to the current online research will probably have more opportunities to learn about coping strategies and receive medical services for post-COVID conditions, as they will also have access to appropriate medical information via the internet. The prognosis of COVID-19 may be worse than the results of this study when a population sample with low ICT literacy and low education level is included. That is, the results of this study may have provided more optimistic data. For future research, it is recommended to include community samples through face-to-face assessment. The final limitation was that we did not investigate the length of time suffering from physical symptoms. Due to this limitation, our results do not clarify the effects of the duration of physical symptoms on mental health.

## Conclusions

The result of our research suggests that post-COVID conditions occur in about 40%. The SARS-CoV-2 infection may cause long- and short-term health hazards and increase the risk of mental disorders. Therefore, medical policy regarding COVID-19 should include long-term clinical practice to address long-term symptoms and mental health risks.

## Data Availability

The datasets analyzed during the current study available from the corresponding author on reasonable request.

## Abbreviations

COVID-19: Coronavirus disease 2019
FCV-19S: Fear of COVID-19 Scale
GAD-7: General Anxiety Disorder-7 -item
ICT: Information and Communication Technology
IES-R: Impact of Event Scale-Revised
PHQ-9: Patient Health Questionnaire-9
PTSD: Post-Traumatic Stress Disorder
SARS-CoV-2: Severe Acute Respiratory Syndrome Coronavirus 2
