# Correction to: Impact of post-COVID conditions on mental health: a cross-sectional study in Japan and Sweden

**DOI:** 10.1186/s12888-022-03953-9

**Published:** 2022-05-06

**Authors:** Kazuki Matsumoto, Sayo Hamatani, Eiji Shimizu, Anton Käll, Gerhard Andersson

**Affiliations:** 1 Research Center for Child Mental Development, Chiba University, Inohana 1-8-1, Chuo-ku, Chiba City, Chiba Japan; 2 Division of Clinical Psychology, Kagoshima University Hospital, Kagoshima, Japan; 3 Department of Behavioural Sciences and Learning, Linköping University, Linköping, Sweden; 4 Research Center for Child Mental Development, University of Fukui, Fukui, Japan; 5 Department of Cognitive Behavioral Physiology, Graduate School of Medicine, Chiba University, Chiba, Japan; 6 Department of Clinical Neuroscience, Karolinska Institute, Stockholm, Sweden

**Correction to: BMC Psychiatry 22, 237 (2022)**

**https://doi.org/10.1186/s12888-022-03874-7**

Following the publication of the original article [1], the authors identified that the funding note was incorrect. The correct funding note is given below.
